# Discovering Alzheimer Genetic Biomarkers Using Bayesian Networks

**DOI:** 10.1155/2015/639367

**Published:** 2015-08-23

**Authors:** Fayroz F. Sherif, Nourhan Zayed, Mahmoud Fakhr

**Affiliations:** ^1^Systems and Computer Department, Electronics Research Institute (ERI), Giza 12622, Egypt; ^2^Systems and Biomedical Department, Faculty of Engineering, Cairo University, Giza 12316, Egypt

## Abstract

Single nucleotide polymorphisms (SNPs) contribute most of the genetic variation to the human genome. SNPs associate with many complex and common diseases like Alzheimer's disease (AD). Discovering SNP biomarkers at different loci can improve early diagnosis and treatment of these diseases. Bayesian network provides a comprehensible and modular framework for representing interactions between genes or single SNPs. Here, different Bayesian network structure learning algorithms have been applied in whole genome sequencing (WGS) data for detecting the causal AD SNPs and gene-SNP interactions. We focused on polymorphisms in the top ten genes associated with AD and identified by genome-wide association (GWA) studies. New SNP biomarkers were observed to be significantly associated with Alzheimer's disease. These SNPs are rs7530069, rs113464261, rs114506298, rs73504429, rs7929589, rs76306710, and rs668134. The obtained results demonstrated the effectiveness of using BN for identifying AD causal SNPs with acceptable accuracy. The results guarantee that the SNP set detected by Markov blanket based methods has a strong association with AD disease and achieves better performance than both naïve Bayes and tree augmented naïve Bayes. Minimal augmented Markov blanket reaches accuracy of 66.13% and sensitivity of 88.87% versus 61.58% and 59.43% in naïve Bayes, respectively.

## 1. Introduction

One of the important study subjects about human genome is the investigation of genetic variants related to complex diseases. Most of these genome-wide association (GWA) studies [1] are aimed to determine genetic variants possibly related to complex diseases [2, 3]. Genetic variants mostly consist of single nucleotide polymorphisms (SNPs), and human genome is estimated to include around 10 million SNPs [4].

A SNP is a single nucleotide site where exactly two (of four) different nucleotides occur in a large percentage of the population. SNPs can contribute to complex disorders in two different ways, either by changing the structure of a specific protein or by changing the abundance of the protein [5]. This is known as the functionality of the SNPs. Genotyping millions of SNPs is highly expensive. For this reason, it is required to obtain a suitable subset of SNPs to accurately represent the rest of SNPs.

A genetic association study aims to find statistical associations between genotypes (genetic variants) and phenotypes (traits or disease states) and thus to identify genetic risk factors [6]. Studies of cases and controls in unrelated individuals are the most commonly used approach for assessing genetic associations of complex diseases since sufficiently large study populations can be easily assembled without the need to enroll also family members of the recruited participants [3].

Alzheimer's disease (AD) is a brain disease identified by slowly progressing memory failure, confusion, poor judgment, and, ultimately, death [7]. It is the most common form of dementia associated with aging. There are two forms of AD, called familial AD and sporadic AD. The rarer form is early-onset familial AD, which typically starts before 65 years of age. The genetic basis of early-onset AD is well proved, and it shows an autosomal dominant inheritance pattern. Most familial cases of AD are accounted for by mutations in one of three genes (amyloid precursor protein gene, presenilin 1, or presenilin 2). Sporadic AD, also called late-onset AD (LOAD), is the commoner form of AD, accounting for approximately 95% of all AD cases. The onset of LOAD symptoms typically occurs after 65 years of age. LOAD has a heritable component but has a more genetically complex mechanism than familial AD [8]. In the past several years, GWASs have identified more than six hundred genes as susceptibility factors, available in AlzGene database. The apolipoprotein E (APOE) gene has been considered as the strongest consistently replicated genetic risk factor for LOAD.

Bayesian learning is a successful method to learn the structure of data in different applications. Here are some reasons why we choose Bayesian methods. Bayesian methods provide several structure learning algorithms. They provide models of causal influence and allow us to explore causal relationships, perform explanatory analysis, and make predictions. Finally, Bayesian networks provide a way to visualize results. As an alternative, machine learning methods, such as Random Forest (RF), have identified potential causal variants on risk for complex diseases like AD [9–11]. However RF obtained poor results in the ADNI genotype dataset. Label propagation (LP) is used to rank SNPs in genome-wide data [12]. When it has been applied to LOAD it performed better than the three control methods in ranking LOAD SNPs. Many studies tried to improve the accuracy of AD diagnosis over the last years [13]. They monitored AD progression and treatment effects using a number of genetic, biochemical [14], and imaging measures [15]. As of yet, none of them has been considered as an ideal AD biomarker. Due to the complexity of AD, other studies combined two or three of these different biomarkers for higher diagnostic accuracy.

Recent studies have been attempted to correlate high-throughput single nucleotide polymorphism (SNP) data with large-scale imaging data [16] or cerebrospinal fluid (CSF) protein levels. Multimodal study combined magnetic resonance imaging (MRI), fluorodeoxyglucose positron emission tomography (FDG-PET) modalities, and CSF biomarker into a multikernel SVM for classifying Alzheimer versus normal samples with high accuracy [17]. Another study [18] added APOE data to the previous markers (structural MRI, FDG-PET, and CSF) to classify Mild Cognitive Impairment- (MCI-) stable and MCI-converter patients using Gaussian process (GP) classification and SVM. Alzheimer's Disease Neuroimaging Initiative (ADNI) database collected and analyzed thousands of brain images, genetic profiles, blood biomarkers, and cerebrospinal fluid biomarkers that are utilized to measure the disease progress or the treatment effects [19].

Many genes have been linked to the disorder. However, only a minority of them are supported by a sufficient level of evidence. Among all SNPs, only SNPs, belonging to the top 10 AD candidate genes listed on the AlzGene database [20] as of April 14, 2011, were selected after the standard quality control (QC) and imputation steps. Our goal was to identify the subset of SNPs strongly associated with Alzheimer's diseases, from the top ranked susceptibility genes, using different supervised learning Bayesian networks structure methods.

The paper is organized as follows.  Section 2 presents the dataset used in this study, introduces Bayesian network, describes four different supervised BN structural learning algorithms, and explores the proposed system.  Section 3 combines the results of each model and compares them. Finally, Section 4 presents our conclusions.

## 2. Material and Methods

Our goal was to apply Bayesian network structure learning (BNSL) to detect Alzheimer's disease potential causal SNPs. Furthermore, identifying SNPs interacted with causal SNPs in addition to the causal SNPs themselves [21].

**Figure 1:**
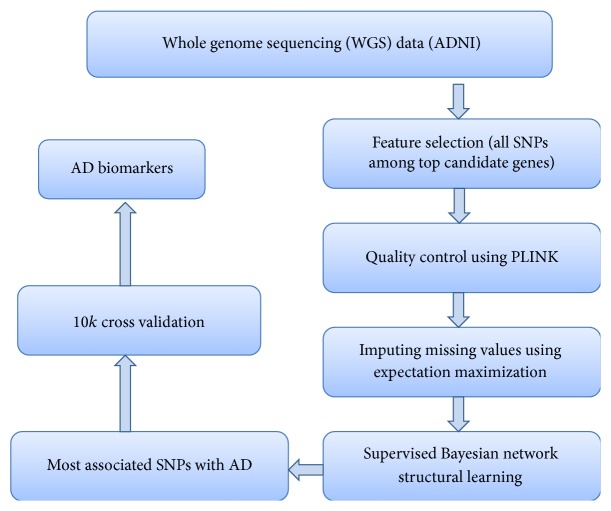
Summary of the proposed system.

The main stages of the proposed system are described in the workflow shown in Figure 1. Start with the whole genome sequencing data, then extract polymorphisms in the top ten genes associated with AD (feature selection), apply quality control based filtering with PLINK, impute the missing values using Expectation Maximization algorithm, use four different Bayesian network structure learning algorithms to get the most associated SNPs with AD, and finally validate the performance of these four BN structures using 10-fold cross validation to reach AD biomarkers.

### 2.1. Datasets

Whole genome sequencing (WGS) data of 812 individuals were obtained from the Alzheimer's Disease Neuroimaging Initiative (ADNI) (As such, the investigators within the ADNI contributed to the design and implementation of ADNI and/or provided data but did not participate in analysis or writing of this report. A complete listing of ADNI investigators can be found at http://adni.loni.usc.edu/wp-content/uploads/how_to_apply/ADNI_Acknowledgement_List.pdf) database. Data used in the preparation of this paper were obtained from the Alzheimer's Disease Neuroimaging Initiative (ADNI) database (http://adni.loni.usc.edu/). The ADNI was launched in 2003 as a public-private partnership, led by Principal Investigator Michael W. Weiner, MD. The primary goal of ADNI has been to test whether serial magnetic resonance imaging (MRI), positron emission tomography (PET), and other biological markers can be combined for early prediction of Alzheimer's disease (AD).

The used subset of the ADNI data includes 282 controls, 442 MCI, and 48 AD as the baseline diagnosis. We selected SNPs belonging to the top ten AD candidate genes listed on the AlzGene database using PLINK program. The total SNP-genotype fields are 496 single SNPs.  Table 1 summarized the top candidate genes used in this study and their identifications: gene name, chromosome number, the number of SNPs among the genes, and their potential pathways [22]. In general, these genes contribute to at least one of three pathways (inflammatory response, endocytosis, and lipid metabolism) all of which have been proposed to play some role in Alzheimer's disease [23].

An initial quality control based filtering with PLINK [24] has been applied for the selected datasets. Firstly individuals with too much missing genotype data (10% missing) and SNPs with a 10% missing genotyping rate (these are the default values) have been excluded.

Subsequently SNPs whose minor allele frequency is less than 0.01 and whose Hardy-Weinberg *p* value is less than 0.001 (the default values in versions prior to 1.04) have been also excluded [25].

Whole genome sequencing (WGS) data used in this study have been gathered from 812 ADNI participants between normal, MCI, and AD. So the phenotype data for the particular patient and this information have been matched with genotype information. We used the phenotype representation of 1 and 2 for normal and AD groups, respectively, according to the baseline exam.

### 2.2. Bayesian Networks

This section explores the Bayesian network approach and its applicability to understand the genetic basis of disease. Bayesian networks are a type of probabilistic graphical models (PGMs) that can represent the conditional dependencies and independencies between a set of random variables via a Directed Acyclic Graph (DAG) [26].

A BN is defined by two models, structural *G* and parametric models Θ, where the structural model *G* of BN has been recognized by nodes *X*_1_,*X*_2_,…,*X*_*n*_ that are representing random variables and arrows between nodes represent a direct dependence among the variables. In particular, an arrow from one node *X*_1_ called parent to another node *X*_2_ called child means that *X*_1_ causes *X*_2_, While lack of edges means independence [27]. The graph *G* encodes independence assumptions, by which each variable *X*_*i*_ is independent of its nondescendants given its parents in *G*. The second component of BN denotes the set of parameters Θ. This parameter *θ* contains the conditional distributions of each node *X*_*i*_ given its parents *π*_*i*_ in *G*, *θ*_*X*_*i*_∣*π*_*i*__ = *p*(*X*_*i*_∣*π*_*i*_). Accordingly,

(1) $$\begin{array}{c} P\left(\;{X_{1},X_{2},\ldots ,X}_{n}\;\right)=\overset{n}{\underset{i=1}{\prod }}\theta_{{X_{i}\;|\;\pi }_{i}}=\overset{n}{\underset{i=1}{\prod }}P\left(\;X_{i}\;|\;\pi_{i}\;\right). \end{array}$$

Most often, the structure and the parameters of Bayesian network are not known a priori and hence need to be learned from data which is called Bayesian network structure learning (BNSL). BNSL has been used in genetic data analysis, classification of disease, and many other areas of biology [28].

Genome-wide association studies (GWASs) aim to identify gene-SNPs involved in human disease or may contribute as a risk factor for developing a complex disease. In order to understand how gene networks contribute to a certain disease, Bayesian networks have been used to represent the relationship between genetic variants and a phenotype (disease status).

The following subsections present different classification algorithms supported by BayesiaLab [29], ordered by their structural complexity. The structural complexity is related to the type and number of dependencies allowed between variables. Four types of structures are presented: naïve Bayes (NB), tree augmented Bayes (TAB), Markov blanket (MB), and minimal augmented Markov blanket (MAMB).

#### 2.2.1. Naïve Bayes Structure (NB)

The least complex structure is the naïve Bayes structure (NB structure), which supposes that predictor variables are conditionally independent given the class. It means ignoring interactions between attributes within individuals of the same class. In naïve Bayes structure all variables are children of the target variable. A Bayesian classifier structure has been created from training data, but this typically requires the probabilities for each variable node given the class variable and the prior probabilities of the class [30].

#### 2.2.2. Tree Augmented Naïve Bayes (TANB)

The augmented naïve Bayesian algorithm begins with an NB structure but relaxes the conditional independence assumption between the child variables. After creating the standard NB structure, a greedy search algorithm has been used to find connections between the child nodes. In tree augmented naïve Bayes (TANB) structure the class variable has no parents and each variable node has at most two parents, one of them is the class variable [30].

#### 2.2.3. Markov Blanket (MB)

It is an algorithm that searches the nodes belonging to the Markov blanket of the target node, that is, fathers, sons, and spouses. The knowledge of the values of each node of this subset of nodes makes the target node independent of all the other nodes. The search of this structure, which is entirely focused on the target node, makes it possible to obtain the subset of the nodes that are really useful much more quickly than other algorithms like naïve Bayesian. Furthermore, this method is a very powerful selection algorithm and is the ideal tool for the analysis of a variable [31].

#### 2.2.4. Minimal Augmented Markov Blanket (MAMB)

Minimal augmented Markov blanket starts with the Markov blanket structure and then uses an unsupervised search to find the probabilistic relations between each of the variables belonging to the Markov blanket. MAMB allows reducing the set of nodes, and it results then in a more accurate target analysis [32].

## 3. Results and Discussion

Bayesian network structural learning has been used to establish a causal relationship or dependency between SNPs in the network and to identify the most efficient path towards AD diagnosis. We introduced a framework for comparing different Bayesian network algorithms to achieve the highest performance improvements. We randomly selected 20% of the dataset as Test Set and consequently the remaining 80% served as our Learning Set. Expectation Maximization algorithm has been used to handle missing values in BN learning. It is an iterative method in which it uses other variables to guess a value (Expectation) and then checks whether that value is the most likely (Maximization). If not, it reguesses more likely values. This repeats until it reaches the most likely value [33]. Although the percentage of the missing data in this study is very small (0.28%), it is better to use EM imputation algorithm than to ignore the problem altogether.

### 3.1. Model Complexity

We have managed network complexity via the Structural Coefficient (SC) parameter. Various experiments for different range values of SC were carried out to find relationships/links between the variables. These experiments indicated that choosing SC value to be 0.25 for MB and MAMB worked much faster and found significant relationships between the variables.

### 3.2. Network Learning

**Figure 2:**
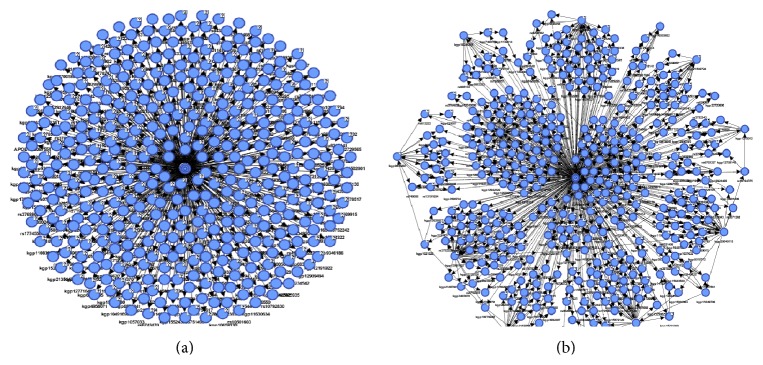
(a) Naïve Bayes structure. (b) Tree augmented naïve Bayes structure.

**Figure 3:**
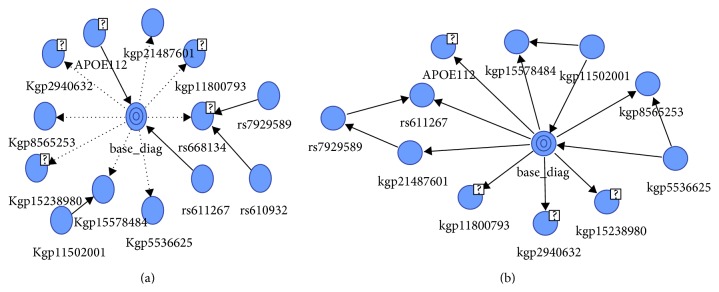
The network structure of (a) Markov blanket algorithm and (b) minimal augmented Markov blanket.

**Figure 4:**
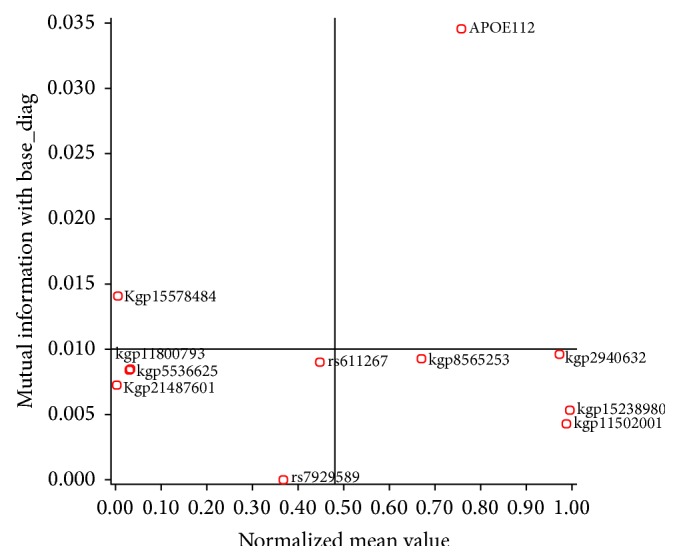
Top related SNPs with Alzheimer' disease using minimal augmented Markov blanket (SNPs kgp11800793 and kgp5536625 overlapped as they have the same mutual information with AD).

We have applied four different supervised algorithms (naïve Bayes, tree augmented naïve Bayes, Markov blanket and minimal augmented Markov blanket) to predict the state of the diagnostic variable, that is, normal or AD. The four resulting Bayesian networks for the classification were shown in Figures 2 and 3, showing both the target node and the predictor SNPs. In the naïve Bayes classifier all variables are included in the model, so the classifier structure is given a priori: complete NB structure. The complete NB classifier structure is shown in Figure 2(a). The accuracy obtained with this classifier in its discrete version is high in some domains. TANB starts from a complete NB structure and continues adding allowed arcs between predictors until the complete TANB structure is formed as shown in Figure 2(b). On the other hand MB and MAMB algorithms identified the most relevant SNPs connected to the disease. The remaining variables are conditionally independent of affected ones.  Figure 3 shows the network structure of the most relevant SNPs that are connected to the disease and resulted from applying Markov blanket and minimal augmented MB algorithms in Figures 3(a) and 3(b), respectively. The figure indicates both the target node (base_diag) and the predictor SNPs (like APOE112, kgp21487601, kgp11800793, etc.). Furthermore, the running time of MB and MAMB is faster than both NB and TANB for the same dataset. These results demonstrate the effectiveness of using BN for identifying AD causal SNPs.  Figure 4 shows the top-correlated SNPs with Alzheimer's disease. SNPs APOE112, kgp21487601 (rs114506298), kgp11800793 (rs113464261), kgp5536625 (rs9331942), kgp15578484 (rs7530069), kgp11502001 (rs73504429), kgp15238980 (rs76306710), kgp8565253 (rs4732729), kgp2940632 (rs4844609), rs792589, and rs611267 are common predictors between MB and MAMB. MB has extra two SNP predictors: rs668134 and rs610932.

SNP APOE112 located in the APOE gene on chromosome 19 presents a significant score of association with AD. SNP APOE112 was the first correlated SNP with AD that resulted from the four Bayesian models. This result confirms that APOE is the highest known AD risk factor. SNP rs769449 located in APOE on chromosome 19 was the second correlated SNP with AD that resulted from both NB and TANB, while kgp15578484 (rs7530069) located in CR1 gene on chromosome 1 was the second correlated SNP with AD that resulted from both MB and MAMB.

Some of the SNPs in our study that were shown to be associated with AD risk have been previously identified in other studies like APOE112, rs4844609, rs769449, rs4732729, rs9331942, rs610932, and rs611267.

Other new SNPs were observed to be significantly associated with Alzheimer's disease. These SNPs are rs7530069, rs113464261, rs114506298, rs73504429, rs7929589, rs76306710, and rs668134. Some other SNPs previously observed to be associated were tested in our study and were not significant. The reason that our results did not include these SNPs was due to an insufficient sample size. Further studies may be needed in larger populations with larger numbers of SNPs.

### 3.3. Model Performance and Evaluation

The overall performance can be expressed as the total precision, which is computed as the total number of correct predictions (true positives + true negatives) divided by the total number of cases in the Test Set. Standard accuracy comparisons were carried out for the four algorithms on all the datasets. Prediction accuracy results, sensitivity, and specificity are reported in Table 2.

The table also indicated the number of predictor SNPs that resulted from each algorithm. For naïve and tree augmented naïve networks a total of 435 distributed SNPs out of 496 SNPs were considered as predictors. However, the number of predictor SNPs reduced to 13 and 11 for Markov blanket and minimal augmented MB, respectively, with higher accuracy.

**Figure 5:**
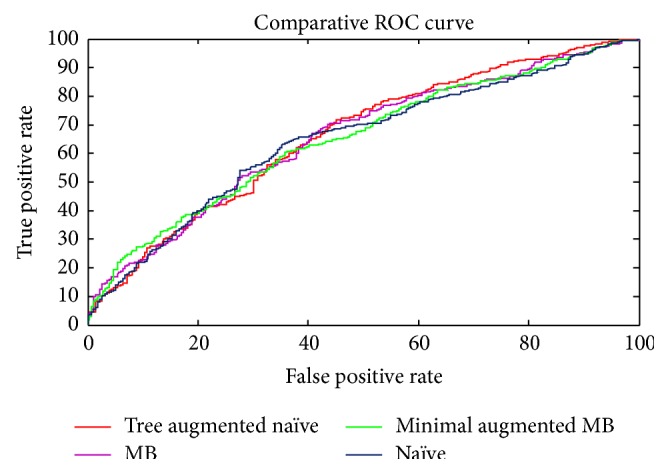
Comparative ROC curve of the four resulting structures.

We evaluated the performance of these four BN structures using 10-fold cross validation. The dataset was randomly partitioned into ten approximately equal sets such that each set had a similar proportion of individuals who developed AD. We applied the algorithms on nine sets taken together as the training data and evaluated the classifier performance on the remaining test data. We repeated this process for each possible test set to obtain an AD prediction for each individual in the dataset. We used the predictions to compute the Receiver Operating Characteristic (ROC) curve which is a widely used measure of classification performance. ROC graphs allowed a broader comparison of classifiers than that available from a single-value metric such as accuracy estimation and may reveal different trends in performance.  Figure 5 represents a comparative ROC curve of the four resulting network structures. In Figure 5, there is reasonable correspondence with the results of Table 2. In general, there is no real difference in performance between any of the classifiers, based on their ROC curves. Tree augmented naïve Bayes appears better than that of naïve Bayes at false positive rate greater than 50%, although TAN had the highest accuracy in Table 2. Interestingly, despite the smaller number of predictors in minimal augmented Markov blanket, the classification performance achieves slightly better performance than other methods. These results were expectable, since the AD genetics is a complex one. The performance of the proposed method may be significantly improved by applying hybrid techniques of Bayesian network with genetic algorithm GA or particle swarm optimization PSO to increase the search efficiency and determine an accurate network. Another machine learning techniques like multifactor dimensionality reduction (MDR), tree based algorithms, or RelifeF filtering may be used to detect associations between SNPs and AD in a higher accuracy, through investigating multiple interactions among SNPs in a case-control study. Finally, using the whole genome sequencing data, not only the top related genes, or adding other modalities like MRI, PET, or CSF biomarkers may significantly improve the prediction accuracy.

## 4. Conclusion

Prediction of complex disease phenotypes from high-throughput genotype data is an emerging research goal. Gene-SNP connectivity and its association with AD can provide critical insights into the underlying mechanisms and identify SNPs that may serve as effective targets for therapeutic intervention. Here we have introduced a framework for the use of four different Bayesian network methods on whole genome sequencing datasets to establish causal relationships among genes and between genes and Alzheimer's disease.

In conclusion, we identified several significant polymorphisms associated with AD, in the APOE, CR1, CD33, CLU, PICALM, and ABCA7 genes. Some of them were previously identified whereas others were novel biomarkers. These results demonstrated the effectiveness of using BN for identifying AD causal SNPs with acceptable accuracy. We hope that our work will facilitate reliable identification of SNPs that are involved in the etiology of Alzheimer's diseases, ultimately supporting timely identification of genomic disease biomarkers, and development of personalized medicine approaches and targeted drug discoveries.

## Figures and Tables

**Table 1:** The top candidate genes and the number of SNPs among each gene.

Gene	Chromosome	Number of SNPs	Potential pathways
APOE	19	6	Cholesterol/lipid metabolism
BIN1	2	101	Endocytic pathways
CLU	8	32	Immune and cholesterol/lipid metabolism
ABCA7	19	36	Cholesterol/lipid metabolism; immune and complement systems/inflammatory response
CR1	1	71	Immune and complement systems/inflammatory response
PICALM	11	138	Endocytic pathways
MS4A6A	11	12	Immune and complement systems/inflammatory response
CD33	19	13	Immune and complement systems/inflammatory response
CD2AP	6	61	Endocytic pathways; immune and complement systems/inflammatory response

**Table 2:** Prediction accuracy results, sensitivity, and specificity for various used algorithms.

Algorithm	Accuracy	Sensitivity	Specificity	Number of SNPs
Naïve Bayes	61.58%	59.43%	65.6%	435
Tree augmented naïve Bayes	64.29%	67.55%	58.16%	435
Markov blanket	65.64%	77.55%	43.26%	13
Minimal augmented Markov blanket	66.13%	88.87%	16.31%	11
